# Exploring the Mechanisms behind the Anti-Tumoral Effects of Model C-Scorpionate Complexes

**DOI:** 10.3390/molecules28145451

**Published:** 2023-07-17

**Authors:** Pedro M. G. Silva, Pedro F. Pinheiro, Sérgio P. Camões, Ana P. C. Ribeiro, Luísa M. D. R. S. Martins, Joana P. G. Miranda, Gonçalo C. Justino

**Affiliations:** 1 Research Institute for Medicines (imed.ULisboa), Faculty of Pharmacy, Universidade de Lisboa, Av. Professor Gama Pinto, 1649-003 Lisboa, Portugal; pmgr.silva@campus.fct.unl.pt (P.M.G.S.); sergiocamoes@campus.ul.pt (S.P.C.); 2 Centro de Química Estrutural—Institute of Molecular Sciences, Instituto Superior Técnico, Universidade de Lisboa, 1049-001 Lisboa, Portugal; pedro.pinheiro@tecnico.ulisboa.pt (P.F.P.); apribeiro@tecnico.ulisboa.pt (A.P.C.R.); 3 Departamento de Engenharia Química, Instituto Superior Técnico, Universidade de Lisboa, 1049-001 Lisboa, Portugal

**Keywords:** C-scorpionate, iron, cobalt, coordination compound, anticancer drug, anti-proliferation, anti-migration, metabolomics

## Abstract

The growing worldwide cancer incidence, coupled to the increasing occurrence of multidrug cancer resistance, requires a continuous effort towards the identification of new leads for cancer management. In this work, two C-scorpionate complexes, [FeCl_2_(*κ*^3^-Tpm)] (**1**) and [Co(*κ*^3^-Tpm^OH^)_2_](NO_3_)_2_ (**2**), (Tpm = hydrotris(pyrazol-1-yl)methane and Tpm^OH^ = 2,2,2-tris(pyrazol-1-yl)ethanol), were studied as potential scaffolds for future anticancer drug development. Their cytotoxicity and cell migration inhibitory activity were analyzed, and an untargeted metabolomics approach was employed to elucidate the biological processes significantly affected by these two complexes, using two tumoral cell lines (B16 and HCT116) and a non-tumoral cell line (HaCaT). While [FeCl_2_(*κ*^3^-Tpm)] did not display a significant cytotoxicity, [Co(*κ*^3^-Tpm^OH^)_2_](NO_3_)_2_ was particularly cytotoxic against the HCT116 cell line. While [Co(*κ*^3^-Tpm^OH^)_2_](NO_3_)_2_ significantly inhibited cell migration in all tested cell lines, [FeCl_2_(*κ*^3^-Tpm)] displayed a mixed activity. From a metabolomics perspective, exposure to [FeCl_2_(*κ*^3^-Tpm)] was associated with changes in various metabolic pathways involving tyrosine, where iron-dependent enzymes are particularly relevant. On the other hand, [Co(*κ*^3^-Tpm^OH^)_2_](NO_3_)_2_ was associated with dysregulation of cell adhesion and membrane structural pathways, suggesting that its antiproliferative and anti-migration properties could be due to changes in the overall cellular adhesion mechanisms.

## 1. Introduction

The World Health Organization (WHO) reported that cancer is the second leading cause of death globally [1,2]. The US National Cancer Institute (Rockville, MD, USA) recognized the existence of over 150 different types of human cancers that can be classified according to the location in the body and the tissue in which they arise [3]. 

Given the adaptive nature of cancer progression, leading to the drug resistance, the molecular toolbox for fighting cancer needs to be constantly expanded, aiming towards the development of novel and effective therapeutics and management approaches [4,5]. 

The family of C-scorpionate metal complexes constitutes an example of coordination compounds extensively employed in organometallic and bioinorganic chemistry, prospective advanced materials, and modelling of active sites of metalloenzymes owing to their molecular design versatility. C-scorpionates are accessible coordination compounds usually formed by tridentate ligands that bind the metal with two in-plane donor atoms, with the third donor site reaching over the plane formed by the metal and the other two donor atoms, resulting in an overall scorpion-like conformation, a distinct feature responsible for the designation of these molecules [6]. 

Several C-scorpionate metal complexes revealed potential as in vitro cytotoxic agents, through intrinsic photonuclease activity via generation and accumulation of reactive oxygen species, with the ligands themselves manifesting antitumor activity [7]. Other studies have been dedicated to the antiproliferative activity of scorpionates, which have also been explored in other clinical treatment approaches, that involve their use as carbon monoxide releasing molecules and photosensitizers, equally relevant areas in cancer therapy research [7]. 

The unique redox properties and electronic structures of cobalt complexes justify their potential to be applied as drug delivery devices, enzyme inhibitors and DNA binding and cleavage agents [8,9]. Some of these cobalt complexes revealed redox-dependent targeting of the tumoral tissues, a feature that contributes to their relevance in anticancer therapy research [9].

**Figure 1:**
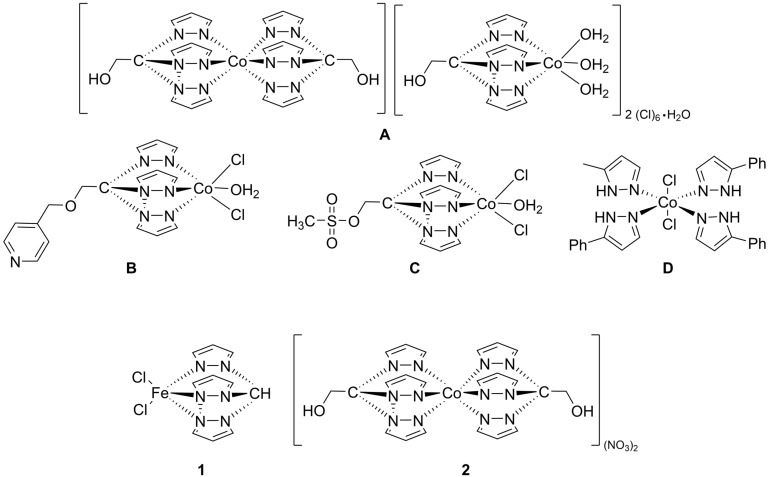
Molecular structure of the scorpionate complexes studied in this work, [FeCl_2_(*κ*^3^-Tpm)] (**1**) and [Co(*κ*^3^-Tpm^OH^)_2_](NO_3_)_2_ (**2**). Complexes **A**–**D** are C-scorpionate complexes with interesting cytotoxicity results.

The antiproliferative activity of three water soluble cobalt C-scorpionates with hydrotris(pyrazol-1-yl)methane (Tpm) ligands (**A**–**C**, Figure 1) has been investigated in the human cancer cell lines HepG2 hepatocellular carcinoma and HCT116 colorectal carcinoma [10]. These complexes revealed moderate cytotoxic effects, leading to a cell viability loss related to an increase in cellular death by apoptosis, but this cytotoxic activity was lower than that induced by cisplatin. **A**, **B**, and **C** exhibited IC_50_ values of 280, 418, and 658 μM towards HCT116, and of 130, 391, and 626 μM towards HepG2 cells, for **A**, **B**, and **C**, respectively, comparing to 4.14 μM for cisplatin. However, these complexes display a much higher water solubility compared to cisplatin. In vitro DNA studies also revealed that two of these cobalt scorpionates promoted double-strand plasmid DNA cleavage [10,11].

Another cobalt complex (a mononuclear tetrakis–phenyl–pyrazolyl non-scorpionate complex, **D**, Figure 1) displayed a higher cytotoxicity in the HCT116 cell line (170 μM) than in the MCF-7 (285 μM) and HepG2 (>500 μM) cell lines [11]. This pyrazolyl Co complex revealed, nonetheless, a very low toxicity towards the normal human in the human cancer cell lines MCF-7 breast carcinoma and HCT116, and in one normal human fibroblast cell line, which was considered a promising result, pointing to a certain specificity in terms of cytotoxic activity of this complex towards carcinoma cells [11]. 

C-scorpionate metal complexes revealed, throughout the last few years, a considerable potential to be employed in various biomedical applications. Specifically, the properties of transition metal ions with pyrazolyl assisted precursors can be taken into advantage in the production of new potential anticancer agents with several mechanisms of action capable of targeting different cancer cells [12,13]. Cobalt complexes, in particular, display the metal in its Co(II) oxidation state, where no relationship between oxidation potential and bioactivity was established [13], and their anti-proliferative activity has been linked to their in vitro induction of double strand breaks [12].

On the other hand, despite the recognition that iron in biology is unique among transition metals due to its widespread use in biological reactions and its abundance in the terrestrial environment (compared to other redox active metals in biology), to date, the biological properties of iron C-scorpionate complexes is unknown. Iron could be both an essential nutrient and potentially toxic to cells, requiring highly sophisticated and complex sets of regulatory approaches to meet the demands of cells while preventing excess accumulation. Therefore, an important aim of this study was to initiate the disclosure of the biological behavior of C-scorpionate iron complexes, namely bearing an iron(II) center.

In particular, two tris(pyrazol-1-yl)methane-type C-scorpionate transition-metal complexes (Figure 1), one neutral, containing an iron(II) center ([FeCl_2_(*κ*^3^-Tpm)], (**1**), Tpm = hydrotris(pyrazol-1-yl)methane) and the other, dicationic, bearing a cobalt(II) center ([Co(*κ*^3^-Tpm^OH^)_2_](NO_3_)_2_, (**2**), Tpm^OH^ = 2,2,2-tris(pyrazol-1-yl)ethanol) were synthetized [10,14] taken into advantage the chemical versatility, high stability, and solubility offered by those ligands, which constitute relevant features that could allow the direct administration of these complexes in several treatment protocols. Similar compounds containing these ligands showed promising catalytic effects for industrial processes, with others exhibiting antitumoral properties that could be exploited for the development of new chemotherapeutic agents [9], which motivated the antiproliferative and the antimigration study of these two previously mentioned C-scorpionate complexes. 

This work aimed to analyze in vitro anti-tumoral properties of the iron ([FeCl_2_(*κ*^3^-Tpm)], **1**) and cobalt ([Co(*κ*^3^-Tpm^OH^)_2_](NO_3_)_2_, **2**) C-scorpionate complexes, through the evaluation of their effects on cell proliferation and migration on two tumoral cell lines, HCT116 (human colorectal carcinoma cells) and B16 (murine melanoma cells) that serve as representative models of colon tumor and melanoma. Colorectal carcinoma constitutes one of the four most common cancer types, being also one of the most lethal [15]. The death rate has been declining, however, throughout the last years, as a result of the increased screening that may help to avert the occurrence of metastatic colorectal cancer. Invasive melanomas correspond to about 1% of all skin cancer cases, being, nevertheless, responsible for most of the skin cancer related deaths [15]. The conventional chemotherapeutic approaches often display a lower efficacy towards these two types of cancer, which reinforces the relevance of exploring other metal complexes for the acquisition of newer and more effective treatments. 

The non-tumoral HaCaT cell line (human spontaneously transformed keratinocytes) was additionally selected to assess the effects of both complexes, to verify if these complexes could reveal more specificity towards cancer-derived cell lines. The effect of **1** and **2** at a metabolome level were also studied to identify altered metabolomic profiles and, subsequently, provide further insights regarding their metabolic effects and mechanisms of action. 

## 2. Results and Discussion

### 2.1. Cytotoxic Activity of the Iron and Cobalt Scorpionates

**Figure 2:**
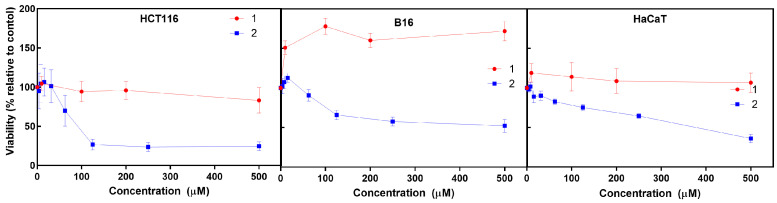
Cell viability of HCT116, B16, and HaCaT cells following 48 h exposure to the iron (**1**, red) and cobalt (**2**, blue) C-scorpionate complexes. Cell viability was determined through the MTS assay. Two independent experiments were performed in octuplicate.

The cytotoxic activity of the iron (**1**) and cobalt (**2**) C-scorpionate complexes was tested using HCT116, B16, and HaCaT cells exposed to 0.01 to 500 μM of **1** and **2** for 48 h and evaluated by the MTS assay (Figure 2), which is based on the reduction of the MTS (3-(4,5-dimethylthiazol-2-yl)-5-(3-carboxymethoxyphenyl)-2-(4-sulfophenyl)-2H-tetrazolium) by viable mammalian cells.

The iron C-scorpionate complex **1** did not reveal a significant toxicity towards the HCT116 and HaCaT cell lines with the tested conditions but led to an increased viability in the B16 cell line, which is accordance with a previous report [16]. In contrast, the cobalt complex **2** revealed a higher cytotoxic effect against the tested cell lines, leading to dose–response curves where the half maximal inhibitory concentration (IC_50_) for each cell line was obtained to provide an indication of the antiproliferative potential of **2**. The IC_50_ for **2** in cell lines HCT116, B16, and HaCaT was approximately 88 µM, 500 µM, and 380 µM, respectively. This higher activity of **2** agrees with previous studies focused on cobalt C-scorpionate complexes that tend to display a superior cytotoxic effect towards this colorectal tumor cell line [6,9]. For comparison, a single dose assay was performed with 1 mM of each ligand—HCT116 cells displayed a 97 ± 9% survival rate when exposed to Tpm and a 94 ± 6% survival rate when exposed to TpmOH. HaCaT cells displayed a 107 ± 11% viability when exposed to Tpm and a 100 ± 10% viability when exposed to TpmOH.

The in vitro cytotoxic and antimigration analyses performed to assess the anticancer capabilities of these C-scorpionate complexes constitute an important step, which serves only as the first in many required to complete the extensive path towards the acquisition of an effective anti-tumoral agent. Cell cultures provide valuable and informative data regarding the effects of particular molecules over specific cellular mechanisms, but these experimental models remain far from being capable of replicating the extensive complexity that surrounds tumoral diseases. The mechanism associated with the decrease in cell viability caused by exposure to the C-scorpionate cobalt complex **2** could possibly be similar to that revealed by other cobalt complexes described in the literature, whose cytotoxic effects arise from the induction of the apoptosis [17,18]. Another cell death mechanism that was recently linked to the antiproliferative effects of a cobalt complex with C-scorpionate precursors involves the induction of autophagy [19], a process that could also be promoted by **2**. Results concerning the cytotoxic effects of **1** and **2** also seem to indicate that the ligands (Tpm and Tpm^OH^, respectively) did not reveal a significant role in this regard [6,9,13]. 

### 2.2. Motogenicity of the Iron and Cobalt C-Scorpionate Complexes

**Figure 3:**
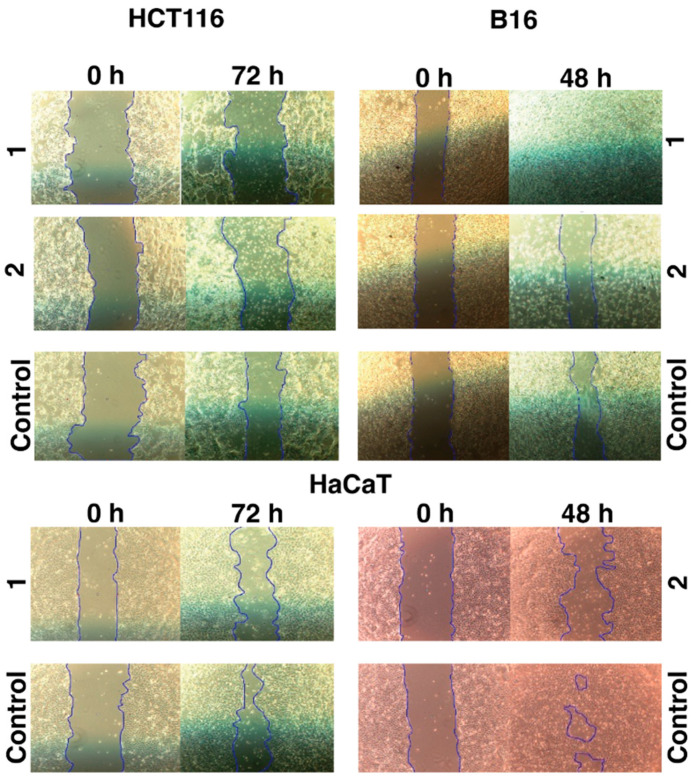
Motogenic effects of the iron **1** and cobalt **2** C-scorpionate complexes on cell migration. The scratch closure corresponds to the percentage of occupied area at each time point, relative to the initial area of the scratch. Culture medium without FBS and culture media containing 2% FBS were used as negative and positive controls, respectively.

The motogenic effects of the tested iron **1** and cobalt **2** C-scorpionate complexes were evaluated through scratch assays at non-toxic complex concentrations (Figure 3). While the iron complex **1** was tested at 200 µM, the cobalt complex **2** was tested at 40 µM. These concentrations allow the assessment of the effect of these complexes on cell migration, while discarding the cytotoxic activity that could otherwise dissimulate their potential antimigration effects.

The iron complex [FeCl_2_(*κ*^3^-Tpm)] (**1**) was able to delay the migration of the cell lines HCT116 and HaCaT, as scratch closure was 7 ± 7% at 72 h for HCT116 (vs. 34 ± 6% for the negative control, with 0% FBS) and 34 ± 4% for HaCaT cells at 72 h (vs. 68 ± 21% for the control). On the B16 cell line, **1** promoted a faster scratch closure, which translates into an increased motogenic effect. Scratch closure in **1**-exposed B16 cells was almost complete at 48 h (99 ± 1 vs. 38 ± 2% for the control) (Figure A2).

The cobalt complex [Co(*κ*^3^-Tpm^OH^)_2_](NO_3_)_2_ (**2**) displayed inhibitory effects in the migration of all the cell lines tested. Scratch closure was 11 ± 7% in HCT116 cells at 72 h (vs. 34 ± 6% for the control), 21 ± 7% in B16 cells at 48 h (vs. 38 ± 2% for the control), and 30 ± 7% in HaCaT cells at 48 h (vs. 75 ± 10% for the control) (Figure A2).

Scratch assays give a clear indication of whether a drug is able to modulate cell migration; the various similarities between chronic wound healing and cancer, highlighted throughout the scientific literature, promoted the use of this wound-healing assay in studies dedicated to cancer cell signaling and behavior [20,21]. The iron complex [FeCl_2_(*κ*^3^-Tpm)] (**1**) revealed the capacity to delay cell migration in the HCT116 and HaCaT cell lines, manifesting a divergent effect in the B16 cell line, where the migration rate was enhanced. On the other hand, the cobalt complex [Co(*κ*^3^-Tpm^OH^)_2_](NO_3_)_2_ (**2**) inhibited the migration of all the cell types used, revealing more pronounced effects over the HaCaT cell line.

Taken together, the antimigration effects of **1** towards HCT116 cell line suggest that this scorpionate complex could still hold some potential regarding its use as an antimetastatic agent. While results with B16 are contradictory, the murine histological origin of the B16 cell line might contribute to this difference when compared with the human cell lines HCT116 and HaCaT. 

By comparison, complex **2** is a much more promising anti-motogenic agent. The cobalt complex displayed a uniform anti-migration effect. HCT116 cells were the most affected by **2** regarding the reduction in viability that was also capable of interfering with their migration, which could support the particular application of this cobalt complex in the treatment of colorectal tumors, where it could be employed as an agent capable of targeting either primary tumoral cells or metastatic cells.

The assays used in this study allowed the observation of the effects of **1** and **2** over the proliferation and migration of tumoral cells, but their potential interference in other relevant tumoral properties, including vascularization, immunomodulation, and the microenvironment, remain uncharted. 

### 2.3. Metabolome-Wide Effect of the Iron and Cobalt Complexes

**Figure 4:**
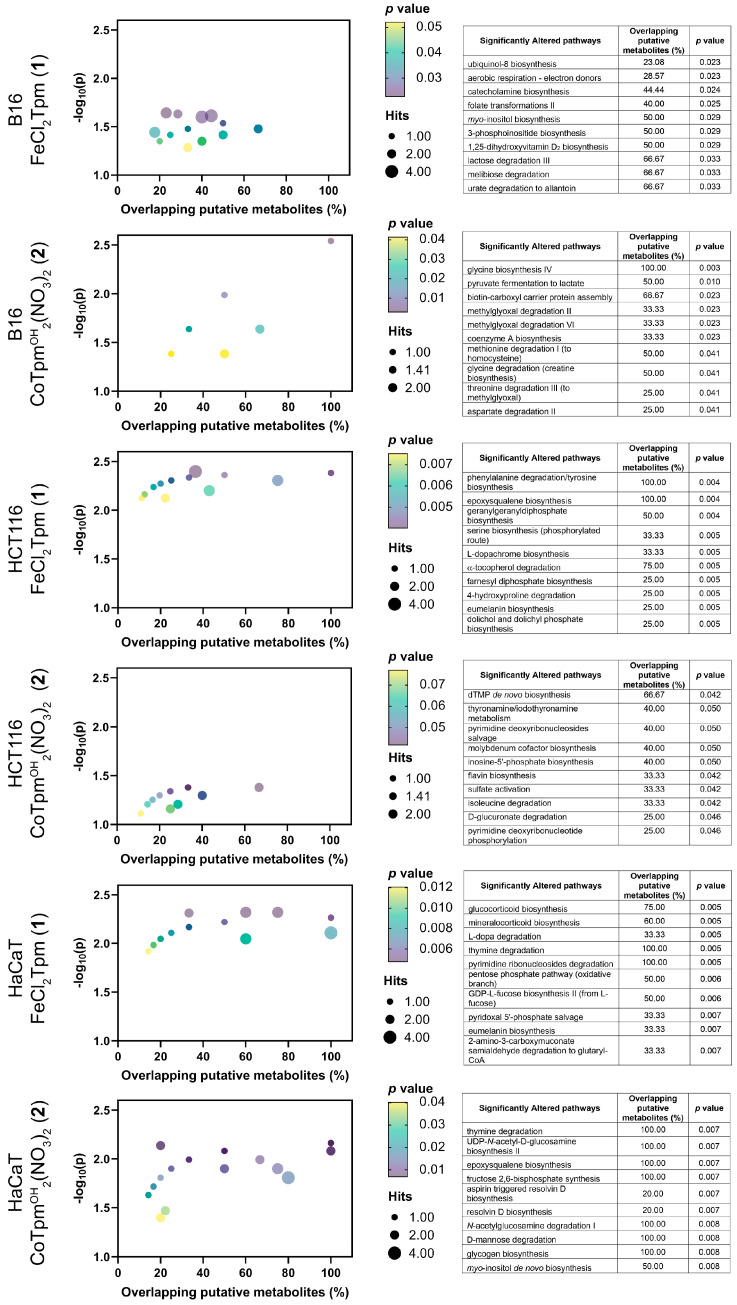
Metabolomics data from cultured cells exposed to the iron, [FeCl_2_(*κ*^3^-Tpm)] (**1**) and [Co(*κ*^3^-Tpm^OH^)_2_](NO_3_)_2_ (**2**) C-scorpionate complexes. The cloud plots on the left show the significantly altered pathways as a function of metabolite overlap and *p* value (color scale) together with the number of overlapping metabolites (circle radius scale). On the right, the tables supply the information regarding the top 10 altered metabolite pathways that display higher significance.

To obtain a deeper knowledge of the cell-wide effects of **1** and **2**, a metabolomics analysis was performed on cells exposed to each drug, at the same concentration used for the scratch assays (200 μM, **1** or 40 μM, **2**). Results were analyzed from an enrichment perspective, aiming to identify pathways significantly dysregulated upon exposure to both complexes (Figure 4). For the assay of the cobalt complex [Co(*κ*^3^-Tpm^OH^)_2_](NO_3_)_2_ (**2**) on HCT116 cells, only data from reverse-phase chromatography yielded significant (*p* < 0.05) enrichment results.

At the metabolome level, the action of both complexes encompasses generic changes in the amino acid and carbohydrate transformation routes, as well as in several cofactor pathways, indicating that these complexes lead to system-wide metabolic changes, in keeping with the previous results from cell viability and cell migration assays.

Regarding the iron complex **1**, specific changes in the tyrosine-associated metabolic biosynthesis processes were observed, namely at the catecholamine, eumelanin, L-dopa, and L-dopachrome pathways. Phenylalanine hydroxylase, tyrosine hydroxylase, and dopamine-b-hydroxylase are iron-dependent enzymes catalyzing various steps in these pathways, and that can be particularly sensitive to iron complexes such as **1**. [FeCl_2_(*κ*^3^-Tpm)] (**1**) is also particularly associated with nucleotide metabolism, including pyrimidines and purines, and appears to modulate glucocorticoid and mineralocorticoid production, associated with the mevalonate-dependent biosynthesis of coenzyme Q10 production. Coenzyme Q10 is the oxidized form of ubiquinol-8, and the biosynthesis of the latter was also found to be affected by **1** in B16 cells [22,23,24].

The metabolomic effect of **1** is also characterized by changes in signal transduction, including the inositol and phosphoinositide pathways, as well as changes in the geranyl and farnesyl phosphate isoprene biosynthesis, involved in the prenylation and membrane anchoring of specific proteins, noticeably signaling Ras proteins [25].

Regarding the tested cobalt complex [Co(*κ*^3^-Tpm^OH^)_2_](NO_3_)_2_ (**2**), specifically altered pathways involve *N*-acetylglucosamine and epoxysqualene biosynthesis, involved in the synthesis of glycolipids and glycoproteins and in the maintenance of membrane integrity, indicating that this [Co(*κ*^3^-Tpm^OH^)_2_](NO_3_)_2_ complex may play an important role in cell integrity and adhesion [26]. 

When evaluated by cell line, distinct effects can be observed, when excluding the carbohydrate, amino acids, and cofactors pathways. The effect of **1** on HCT116 cells is predominantly associated with changes involving signaling and structural lipids. In HaCaT cells, these pathways are accompanied by other signal transduction events, involving glucocorticoids and mineralocorticoids; HaCaT also display changes in several glycan-associated pathways. In the case of the B16 cells, exposure to the iron complex (**1**) led to significant changes in several lipid and sugar signaling levels.

Taken together, metabolomics indicates that the major actions of these two complexes are at the signaling level, particularly in pathways involving lipids and, to some extent, sugar, and tyrosine.

The localization of these effects to the cellular membrane strongly suggest that adhesion mechanisms are a target of this cobalt complex. The phosphoinositide signaling pathways are particularly relevant in cell motility [27], and the observed *myo*-inositol biosynthesis dysregulation supports the role of **2** as a modulator of cell adhesion and motility. Additional studies could include the use of other tumoral and normal (nonmalignant) cell lines, to confirm their specificity towards cancer cells. Other studies could involve cell invasion assays, where the effects of these complexes on the cell capacity to bypass specific barriers may be assessed.

## 3. Conclusions

This work focused on the preliminary assessment of antitumoral properties displayed by two C-scorpionate complexes with different metal centers, by evaluating their antiproliferative and antimigration capabilities, and by assessing their metabolome-wide effects on the melanoma B16 and colon carcinoma HCT116 cell lines vs. the non-tumoral HaCaT cell line.

The tested iron complex [FeCl_2_(*κ*^3^-Tpm)] (**1**) did not display significant toxicity towards any cell line; in fact, it led to increased B16 viability. On the contrary, the [Co(*κ*^3^-Tpm^OH^)_2_](NO_3_)_2_ (**2**) complex displayed a higher cytotoxic effect against the tested cell lines, with IC_50_ values of 88 µM and 500 µM towards the HCT116 and B16 cell lines, respectively, which compare to an IC_50_ of 380 µM towards the HaCaT cell line. Similarly, complex **2** was able to markedly inhibit the migration of all cell lines, while **1** displayed a much lower activity [16]. Moreover, the metabolomics effects observed in the present work suggest that the action of the [Co(*κ*^3^-Tpm^OH^)_2_](NO_3_)_2_ (**2**) C-scorpionate complex occurs via changes at glycan pathways and lipid and carbohydrate signaling pathways [27].

In conclusion, the cobalt scorpionate [Co(*κ*^3^-Tpm^OH^)_2_](NO_3_)_2_ (**2**) displayed a promising antiproliferative and anti-motogenic activity. Previous studies indicated that Co complexes can be more active that complexes with other metals [10,13,28]. These results are supported by the metabolomics alterations observed in cell adhesion- and membrane-structure-associated pathways. Taken together, these results suggest that [Co(*κ*^3^-Tpm^OH^)_2_](NO_3_)_2_ can be a promising scaffold for the development of anti-cancer drugs, requiring further exploration from a structural diversity point of view, geared towards the maximization of their in vitro activity. Furthermore, a strict elucidation of the pathways involved in the activity of these compounds is required to better understand the mechanisms underlying the biological activity of these compounds.

## 4. Materials and Methods

### 4.1. Reagents

Cell culture media (McCoy’s 5A and DMEM), FBS (fetal bovine serum), trypsin (0.25%), penicillin-streptomycin solution, and DMSO (dimethyl sulfoxide) were purchased from Sigma-Aldrich (Barcelona, Spain). D-glucose was acquired from AppliChem (Barcelona, Spain). CellTiter 96^®^AQueous Non-Radioactive Cell Proliferation Assay (used for the cellular viability assays) was purchased from Promega (Lisbon, Portugal).

### 4.2. Preparation of Scorpionate Solutions

**Figure A1:**
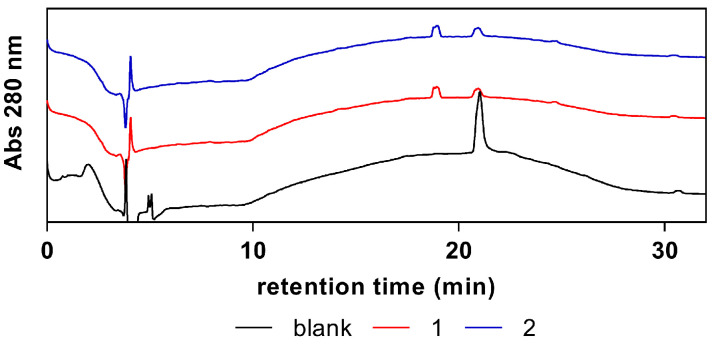
Purity assessment of the synthesized complexes (**1**) and (**2**).

**Figure A2:**
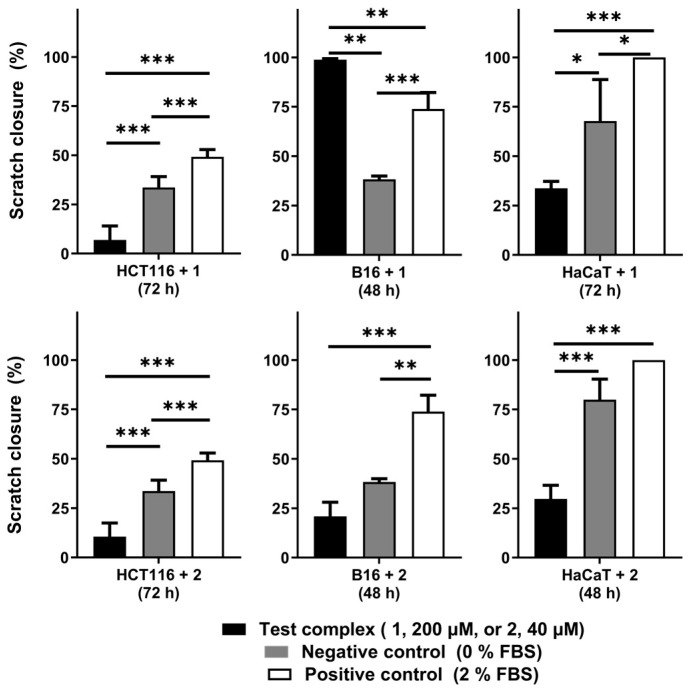
Effect of complexes (**1**) and (**2**) on cell migration. Results shown (average ± standard deviation) are the average of three independent experiments, each performed in quadruplicate. *, *p* < 0.05; **, *p* < 0.01; ***, *p* < 0.001.

The [FeCl_2_(*κ*^3^-Tpm)] (**1**) and [Co(*κ*^3^-Tpm^OH^)_2_](NO_3_)_2_ (**2**) scorpionate complexes (Figure 1) were prepared as described in the literature [10,14], and were stored in the form of refined powder at room temperature. The purity of each compound was determined by RP-HPLC (reverse-phase high-performance liquid chromatography) analysis (Figure A1). HPLC analyses were performed in a modular HPLC system composed of a Varian ProStar 410 autosampler, two 210-LC chromatography pumps, and a ProStrar 325 UV detector (Varian, Inc., Palo Alto, CA, USA). Data acquisition and processing were performed using Varian MS Control 6.9.6 software. Samples were prepared in water from concentrated solutions and contained 1 mM of test compound and 0.5 mM of internal standard (4-hydroxytoluene). Samples (5 µL) were injected onto the column via a Rheodyne injector (Rheodyne LLC., IDEX Corp., Lake Forest, IL, USA) with a 100 µL loop in the µL pickup injection mode. Separations were conducted at room temperature, using a ThermoFisher Scientific BDS Hypersil C18 (250 mm × 4.6 mm, 5 µm) reversed phase column and a 1 mL/min flow rate. The mobile phase consisted of 0.1% (*v*/*v*) formic acid in water (A) and acetonitrile (B). The following elution gradient was used: 0–2 min isocratic 5% B, 2–22 min linear gradient to 100% B, 22–28 min isocratic 100% B, 28–30 min linear gradient to 5% B, 30–32 min isocratic 5% B. Chromatograms were recorded at 280 nm. 

### 4.3. HCT116, B16, and HaCaT Cell Culturing

HCT116 (human colorectal carcinoma) and B16 (human melanoma) cell lines were obtained from American Type Culture Collection, while the HaCaT cell line (spontaneously immortalized keratinocyte) was obtained from Cell Line Services.

Cells were seeded in appropriate cell culture T-flasks using McCoy’s 5A medium for HCT116 cells or DMEM supplemented with 4.5 g/L D-(+)-Glucose (DMEM High Glucose) for B16 and HaCaT cells, supplemented with 10% FBS, and incubated at 37 °C in a humidified atmosphere with 5% CO_2_. Cell passages were performed every 3 to 4 days, depending on cell confluence, by Trypsin/EDTA 0.25% incubation for 5 min, after which the cells were centrifuged at 200× *g* for 5 min, resuspended in the recommended culture medium. Cell counting was performed using a hemocytometer, and their viability was assessed through trypan blue staining.

### 4.4. Cytotoxicity Assays

The HCT116, B16, and HaCaT cells were seeded into 96-well plates at a density of 3.0 × 10^4^ cells/cm^2^ with the respective recommended culture medium with 10% FBS. After 24 h, the culture medium was removed from the wells and replaced with 200 µL of complete culture medium supplemented with varying concentrations of either complex (**1** or **2**), previously dissolved in H_2_O. The positive control consisted in cells cultured in complete culture medium with FBS, the solvent control corresponded to culture medium supplemented with water, and the negative control consisted of culture medium supplemented with DMSO. After an incubation period of 48 h, the cytotoxicity of **1** and **2** was assessed through the MTS assay. 

Cells were washed with PBS and incubated in 100 µL of DMEM with 10% FBS and 20 µL of MTS for 2 h in 96-well plates. Absorbance measured at 690 nm and 490 nm on a SPECTROstar Omega Microplate Reader. At least two independent experiments were performed, in quadruplicate. Results were expressed as percentage based on the solvent control, which, in this assay, was considered to correspond to 100% of cellular viability/proliferation.

### 4.5. Scratch Assays

The effect of the iron [FeCl_2_(*κ*^3^-Tpm)] (**1**) and cobalt [Co(*κ*^3^-Tpm^OH^)_2_](NO_3_)_2_ (**2**) C-scorpionate complexes on the migration of HCT116, B16, and HaCaT cells was evaluated by scratch assays. Cells were cultivated in 24-well plates at a density of 6.0 × 10^4^ cells/cm^2^ using the recommended culture media with 10% FBS. When confluence reached approximately 80%, scratches of about 0.5 mm in width were performed on the cell monolayer. After scratching, cells were washed with PBS and maintained in a final volume of 500 µL of recommended culture media without FBS, supplemented with either **1** or **2**, at non-cytotoxic concentrations. As negative control, culture medium without FBS was used, while the positive control consisted of culture medium containing 2% FBS (a known motogenic factor). In this assay, the negative control translated the natural cell migration. At least two independent experiments were performed, in triplicate. 

Digital photographs of the scratches were taken at several time points (0, 2 h, 16 h, 24 h, 40 h, 48 h, and 72 h post-scratch) at an amplification of 4× on an Olympus CK30 microscope. Cellular migration was assessed using Motic Images Version 3.0 software, where the area of the scratches was measured to calculate scratch closure, given as the percentage of occupied area at each time point, relative to the initial area of the scratch.

### 4.6. Statistical Analysis 

All data were obtained from at least two independent experiments and were expressed as average ± standard deviation (SD). GraphPad Prism software was used to obtain the half maximal inhibitory concentration, IC_50_, and to perform statistical analysis. The two-way ANOVA with multiple comparisons test was used for the in vitro cytotoxicity and scratch assays. *p*-values were represented for statistically significant results.

### 4.7. Metabolomic Analyses

HCT116, B16, and HaCaT cells were cultivated into T-25 flasks at a density of 2.0 × 10^4^ cells/cm^2^ using the recommended culture medium supplemented with 10% FBS. After *ca*. 24 h, culture media was replaced with 3 mL of recommended culture media without FBS and supplemented with 1% Pen-Strep and either **1** or **2**, using the same concentrations that were tested in the scratch assays. The negative control consisted of cells maintained in culture medium without FBS. 

Cells were cultured for 48 h and prepared for analysis as routinely for adherent cells [29]. Briefly, cells were harvested by trypsinization, washed with PBS, and centrifuged at 400× *g* for 5 min The pellets were stored at −80 °C overnight. Frozen cell pellets were resuspended in 500 µL of cold extraction solvent (acetonitrile/methanol/water 2:2:1 *v*/*v*/*v*), frozen in liquid nitrogen for 3 min, and thawed on ice. The freeze–thaw process was repeated 3 times, and samples were centrifuged at 14,000× *g* for 15 min at 4 °C.

An untargeted metabolomics approach was employed to provide a comprehensive analysis of all the metabolites that were significantly altered in the samples exposed to either the iron complex [FeCl_2_(*κ*^3^-Tpm)] (**1**) or the cobalt complex [Co(*κ*^3^-Tpm^OH^)_2_](NO_3_)_2_ (**2**) vs. the control samples, with the additional identification of the altered metabolic pathways associated with those metabolites. High-resolution mass spectrometry (HRMS) analyses were performed using an Elute UHPLC system composed of an Elute UHPLC HPG 1300 pump with two pairs of serial-coupled, individually controlled, linear drive pump heads, an Elute autosampler, and an Elute column CSV preheated oven, coupled to an Impact II QqTOF mass spectrometer with an electrospray ion source (Bruker Daltonics GmbH & Co., Bremen, Germany) (UHPLC-ESI-HRMS). Data acquisition was performed with in-house optimized methods.

Metabolites were separated by reverse-phase UHPLC on a Luna 2.5 µm C18(2)-HST column (100 Å, 150 × 2 mm, Phenomenex, Torrance, CA, USA) at a constant temperature of 40 °C, using a gradient elution at a flow rate of 250 μL/min (mobile phase A: 0.1% (*v*/*v*) formic acid in water; mobile phase B: 0.1% (*v*/*v*) formic acid in acetonitrile): 0.0–0.5 min, 0% B; 0.5–1.5 min, 0 to 20% B; 1.5–4.0 min, 20 to 60% B; 4.0–6.0 min, 60 to 100% B; 6.0–9.0 min, 100% B; 9.0–10.0 min, 100 to 0% B, followed by a 5 min column re-equilibration step. For hydrophilic interaction liquid chromatography (HILIC), an XBridge BEH Amide XP Column (130 Å, 2.5 µm, 150 × 2.1 mm, Waters) was used at a constant temperature of 40 °C. With a flow rate of 250 μL/min, a gradient elution of 10 mM ammonium acetate in water containing 0.1% (*v*/*v*) acetic acid (A) and 10 mM ammonium acetate in acetonitrile containing 2% (*v*/*v*) water and 0.1% (*v*/*v*) acetic acid (B) was applied: 0–2 min, 90% B; 2–6 min, 90 to 70% B; 6–9 min, 70 to 30% B; 9–13 min, 30% B; 13–18 min, 30 to 90% B, followed by a 4 min column re-equilibration step.

High resolution mass spectra were acquired in both ionization modes, with the following acquisition parameters: capillary voltage, 4.5 kV (ESI+) or 3 kV (ESI−); end plate offset, 500 V; nebulizer, 2.0 bar; dry gas (N_2_) flow, 8.0 L/min; dry heater temperature, 220 °C. The tune parameters were set according to: transfer funnel 1/2 RF power (150/200 Vpp), hexapole RF power (50 Vpp), ion energy (4.0 eV), low mass (90 *m/z*), collision energy (7.0 eV), collision RF power (650 Vpp), transfer time (80 μs), pre-pulse storage (5 μs). Spectral acquisition was performed with an absolute threshold of 25 counts per 1000. Mass spectrometer parameters were set as above, except for a dry heater temperature at 220 °C. MS/MS spectra, with an *m/z* scan range from 70 to 1000, were acquired with a 3.00 Hz spectra rate. Thresholds for auto MS/MS were set at 20 counts per 1000, cycle time of 3.0 s with exclusion after 3 spectra and release after 1.00 min. Three full scans and one auto MS/MS scan were performed for each sample, using both positive and negative ionization modes.

The acquired MS data were processed with Data Analysis (version 5.1) (Bruker Daltoniks, Billerica, MA, USA). Raw data were converted to mzXML using ProteoWizard MSConvert [30,31,32,33] and processed on XCMS v3.7.1. Pairwise analysis of data from elution with both LC columns and both ionization modes were then integrated through a multimodal analysis [34], using a 0.05 *p*-value cutoff. 

## Data Availability

Raw MS data, corresponding to the metabolomics study presented, are openly available in Mendeley Data at http://dx.doi.org/10.17632/z2nb649hbz.1, http://dx.doi.org/10.17632/254vj2jgkc.1, and http://dx.doi.org/10.17632/p7rsc48hpt.1 (accessed on 10 July 2023).

## Sample Availability

Samples of the compounds **1** and **2** are available from the authors.

## Appendix A

The purity of the synthesized complexes was confirmed by HPLC, using 4-hydroxytoluene as an internal standard (Figure A1). Only peaks attributed to the scorpionate complexes (at *ca*. 18 min) or to the internal standard (shown inside the dotted box) are shown. Results from the migration assays are given in Figure A2.

## References

[1] Grosso D., Aljurf M., Gergis U., Aljurf M., Majhail N.S., Koh M.B.C., Kharfan-Dabaja M.A., Chao N.J. (2022). Building a Comprehensive Cancer Center: Overall Structure. The Comprehensive Cancer Center—Development, Integration, and Implementation.

[2] Mattiuzzi C., Lippi G. (2019). Current Cancer Epidemiology. J. Epidemiol. Glob. Health.

[3] A to Z List of Cancer Types—NCI. https://www.cancer.gov/types.

[4] Bousbaa H. (2021). Novel Anticancer Strategies. Pharmaceutics.

[5] Holohan C., Van Schaeybroeck S., Longley D.B., Johnston P.G. (2013). Cancer Drug Resistance: An Evolving Paradigm. Nat. Rev. Cancer.

[6] Andrade M.A., Martins L.M.D.R.S. (2019). Novel Chemotherapeutic Agents—The Contribution of Scorpionates. Curr. Med. Chem..

[7] Silva F., Fernandes C., Campello M.P.C., Paulo A. (2017). Metal Complexes of Tridentate Tripod Ligands in Medical Imaging and Therapy. Polyhedron.

[8] Pettinari C., Marchetti F., Lupidi G., Quassinti L., Bramucci M., Petrelli D., Vitali L.A., Guedes Da Silva M.F.C., Martins L.M.D.R.S., Smoleński P. (2011). Synthesis, Antimicrobial and Antiproliferative Activity of Novel Silver(I) Tris(Pyrazolyl)Methanesulfonate and 1,3,5-Triaza-7-Phosphadamantane Complexes. Inorg. Chem..

[9] Martins L.M.D.R.S., Pombeiro A.J.L. (2016). Water-Soluble C-Scorpionate Complexes—Catalytic and Biological Applications. Eur. J. Inorg. Chem..

[10] Silva T.F.S., Martins L.M.D.R.S., Guedes Da Silva M.F.C., Fernandes A.R., Silva A., Borralho P.M., Santos S., Rodrigues C.M.P., Pombeiro A.J.L. (2012). Cobalt Complexes Bearing Scorpionate Ligands: Synthesis, Characterization, Cytotoxicity and DNA Cleavage. Dalton Trans..

[11] Munteanu C.R., Suntharalingam K. (2015). Advances in Cobalt Complexes as Anticancer Agents. Dalton Trans..

[12] Roy S., Patra A.K., Dhar S., Chakravarty A.R. (2008). Photosensitizer in a Molecular Bowl and Its Effect on the DNA-Binding and -Cleavage Activity of 3d-Metal Scorpionates. Inorg. Chem..

[13] Silva T.F.S., Martins L.M.D.R.S., Guedesdasilva M.F.C., Kuznetsov M.L., Fernandes A.R., Silva A., Pan C.J., Lee J.F., Hwang B.J., Pombeiro A.J.L. (2014). Cobalt Complexes with Pyrazole Ligands as Catalyst Precursors for the Peroxidative Oxidation of Cyclohexane: X-Ray Absorption Spectroscopy Studies and Biological Applications. Chem. Asian J..

[14] Silva T.F.S., Alegria E.C.B.A., Martins L.M.D.R.S., Pombeiro A.J.L. (2008). Half-Sandwich Scorpionate Vanadium, Iron and Copper Complexes: Synthesis and Application in the Catalytic Peroxidative Oxidation of Cyclohexane under Mild Conditions. Adv. Synth. Catal..

[15] Viale R.P.H. (2020). The American Cancer Society’s Facts & Figures: 2020 Edition. J. Adv. Pract. Oncol..

[16] Pahonțu E., Proks M., Shova S., Lupașcu G., Ilieș D.-C., Bărbuceanu Ș.-F., Socea L.-I., Badea M., Păunescu V., Istrati D. (2019). Synthesis, characterization, molecular docking studies and in vitro screening of new metal complexes with Schiff base as antimicrobial and antiproliferative agents. Appl. Organomet. Chem..

[17] Alghamdi N.J., Balaraman L., Emhoff K.A., Salem A.M.H., Wei R., Zhou A., Boyd W.C. (2019). Cobalt(II) Diphenylazodioxide Complexes Induce Apoptosis in SK-HEP-1 Cells. ACS Omega.

[18] Verma P.K., Singh R.K., Kumar S., Shukla A., Kumar S., Gond M.K., Bharty M.K., Acharya A. (2022). Cobalt (III) complex exerts anti-cancer effects on T cell lymphoma through induction of cell cycle arrest and promotion of apoptosis. Daru.

[19] Das K., Datta A., Frontera A., Wen Y.S., Roma-Rodrigues C., Raposo L.R., Fernandes A.R., Hung C.H. (2020). Zn(II) and Co(II) Derivatives Anchored with Scorpionate Precursor: Antiproliferative Evaluation in Human Cancer Cell Lines. J. Inorg. Biochem..

[20] MacCarthy-Morrogh L., Martin P. (2020). The Hallmarks of Cancer Are Also the Hallmarks of Wound Healing. Sci. Signal..

[21] Pratt S.J.P., Hernández-Ochoa E.O., Lee R.M., Ory E.C., Lyons J.S., Joca H.C., Johnson A., Thompson K., Bailey P., Lee C.J. (2018). Real-Time Scratch Assay Reveals Mechanisms of Early Calcium Signaling in Breast Cancer Cells in Response to Wounding. Oncotarget.

[22] Shukla S., Dubey K.K. (2018). CoQ10 a Super-Vitamin: Review on Application and Biosynthesis. 3 Biotech.

[23] Ponting C.P. (2001). Domain Homologues of Dopamine Beta-Hydroxylase and Ferric Reductase: Roles for Iron Metabolism in Neurodegenerative Disorders?. Hum. Mol. Genet..

[24] Fitzpatrick P.F. (2023). The Aromatic Amino Acid Hydroxylases: Structures, Catalysis, and Regulation of Phenylalanine Hydroxylase, Tyrosine Hydroxylase, and Tryptophan Hydroxylase. Arch. Biochem. Biophys..

[25] Palsuledesai C.C., Distefano M.D. (2015). Protein Prenylation: Enzymes, Therapeutics, and Biotechnology. ACS Chem. Biol..

[26] Chen Q., Tan Z., Guan F., Ren Y. (2020). The Essential Functions and Detection of Bisecting GlcNAc in Cell Biology. Front. Chem..

[27] Gumbiner B.M. (1996). Cell Adhesion: The Molecular Basis of Tissue Architecture and Morphogenesis. Cell.

[28] Kumbar M., Patil S.A., Kinnal S.M., Jawoor S.S., Shettar A. (2019). Scorpionate Ligand Derived from 1-Amino-9H-Fluoren-9-Ol and Its Metal (II) Complexes as Potential Anticancer Agents. Chem. Data Collect..

[29] Marques C.F., Justino G.C. (2023). An Optimised MS-Based Versatile Untargeted Metabolomics Protocol. Separations.

[30] Chambers M.C., MacLean B., Burke R., Amodei D., Ruderman D.L., Neumann S., Gatto L., Fischer B., Pratt B., Egertson J. (2012). A Cross-Platform Toolkit for Mass Spectrometry and Proteomics. Nat. Biotechnol..

[31] Gowda H., Ivanisevic J., Johnson C.H., Kurczy M.E., Benton H.P., Rinehart D., Nguyen T., Ray J., Kuehl J., Arevalo B. (2014). Interactive XCMS Online: Simplifying Advanced Metabolomic Data Processing and Subsequent Statistical Analyses. Anal. Chem..

[32] Tautenhahn R., Patti G.J., Rinehart D., Siuzdak G. (2012). XCMS Online: A Web-Based Platform to Process Untargeted Metabolomic Data. Anal. Chem..

[33] Forsberg E.M., Huan T., Rinehart D., Benton H.P., Warth B., Hilmers B., Siuzdak G. (2018). Data Processing, Multi-Omic Pathway Mapping, and Metabolite Activity Analysis Using XCMS Online. Nat. Protoc..

[34] Huan T., Palermo A., Ivanisevic J., Rinehart D., Edler D., Phommavongsay T., Benton H.P., Guijas C., Domingo-Almenara X., Warth B. (2018). Autonomous Multimodal Metabolomics Data Integration for Comprehensive Pathway Analysis and Systems Biology. Anal. Chem..

